# Recurrent Acute-on-Chronic Hyponatraemia: A Diagnostic Odyssey Involving Drug-Induced Syndrome of Inappropriate Antidiuretic Hormone Secretion (SIADH), Chronic Cannabis Use, and Occult Lung Adenocarcinoma

**DOI:** 10.7759/cureus.113415

**Published:** 2026-07-26

**Authors:** Rawan Alhashimi, Zin Tun, Erol Agha

**Affiliations:** 1 Internal Medicine, Cambridge University Hospitals NHS Foundation Trust, Cambridge, GBR; 2 Diabetes and Endocrinology, Cambridge University Hospitals NHS Foundation Trust, Cambridge, GBR; 3 Psychiatry, Change Grow Live, Peterborough, GBR

**Keywords:** acute confusion, adenocarcinoma lung, chronic cannabis use, drug-induced siadh, excessive thirst, profound hyponatremia, psychogenic polydipsia, selective serotonin reuptake inhibitor (ssri), syndrome of inappropriate secretion of antidiuretic hormone (siadh)

## Abstract

Hyponatraemia is a common clinical presentation and one of the most frequently encountered electrolyte abnormalities in hospitalised patients, with syndrome of inappropriate antidiuretic hormone secretion (SIADH) representing a major underlying cause. Clinical manifestations are variable, with confusion being a common presenting feature. While hyponatraemia is often readily identified and managed, recurrent or complex presentations may reveal multiple coexisting aetiologies, including malignancy, medication-related causes, substance misuse, and neuropsychiatric disorders. Establishing the underlying cause can be challenging, particularly when initial investigations appear to provide a satisfactory explanation for the presenting episode. This case highlights the importance of ongoing diagnostic reassessment in patients with recurrent hyponatraemia and confusion, as well as the need to consider multifactorial contributors when the clinical course is not fully explained by a single diagnosis.

## Introduction

Hyponatraemia, defined as a serum sodium concentration below 135 mmol/L, is one of the most common electrolyte abnormalities encountered in hospitalised patients and most frequently presents as hypotonic hyponatraemia [1]. Clinical manifestations range from mild, non-specific symptoms to severe neurological complications, including confusion, ataxia, seizures, and reduced consciousness. The underlying pathophysiology is often complex and requires careful evaluation to identify both the immediate cause and any contributing factors.

Antidiuretic hormone (ADH) plays a central role in fluid homeostasis by regulating renal water retention in response to changes in plasma osmolality and effective circulating volume [2]. ADH binds to vasopressin V2 receptors on principal cells of the renal collecting ducts, promoting insertion of aquaporin-2 water channels into the apical membrane, thereby increasing water reabsorption and concentrating the urine. Physiological ADH secretion occurs in states of hypovolaemia, such as gastrointestinal fluid loss, haemorrhage, or intravascular depletion secondary to third spacing, as seen in heart failure, cirrhosis, or systemic infection, where water conservation is necessary to maintain circulatory stability. In contrast, inappropriate ADH secretion may occur despite a euvolaemic state, resulting in the syndrome of inappropriate antidiuretic hormone secretion (SIADH), a major cause of euvolaemic hypotonic hyponatraemia [2].

SIADH is among the most common causes of hyponatraemia in hospitalised patients and may arise secondary to pulmonary disease, malignancy, medications, surgery, or endocrine abnormalities [2]. However, hyponatraemia is frequently multifactorial, particularly in older adults, in whom coexisting medical conditions, polypharmacy, and impaired water excretion may all contribute simultaneously to sodium imbalance [3]. Consequently, recurrent or inadequately explained hyponatraemia should prompt reassessment of the initial diagnosis and consideration of alternative or additional aetiologies.

We present a case of recurrent acute-on-chronic hyponatraemia initially attributed to fluoxetine-induced SIADH, which subsequently led to the diagnosis of early-stage lung adenocarcinoma. Further recurrent admissions revealed significant chronic cannabis use associated with excessive fluid intake and recurrent vomiting, raising the possibility of cannabis-associated thirst with excessive free-water intake and possible cannabinoid hyperemesis syndrome.

## Case presentation

A 73-year-old woman with a past medical history of type 2 diabetes mellitus, essential hypertension, cerebrovascular disease, and depression was referred to the emergency department by her general practitioner with acute confusion, nausea, vomiting, and acute-on-chronic hyponatraemia after the recent bereavement of a close family member.

Initial assessment demonstrated acute confusion with a Glasgow Coma Scale score of 14/15. She appeared cachectic, with evidence of self-neglect. Her blood pressure was 179/80 mmHg, heart rate 83 beats/min, respiratory rate 20 breaths/min, temperature 36.6°C, and oxygen saturation 97% on room air. Her ECG demonstrated normal sinus rhythm at 80 beats/min with normal PR and QRS intervals and a QTc of 445 ms. She was clinically euvolaemic, with normal cardiovascular, respiratory, and abdominal examinations. Neurological examination was notable for significant ataxia without focal neurological deficits.

Initial laboratory investigations demonstrated profound isolated hyponatraemia (serum sodium 116 mmol/L; baseline 128-130 mmol/L since 2020). The remaining blood tests were largely unremarkable, apart from mild anaemia, borderline low serum magnesium, and a transiently elevated lactate that resolved following fluid resuscitation (Table 1). Further investigations demonstrated a low serum osmolality (239 mOsm/kg) and inappropriately concentrated urine (urine osmolality 176 mOsm/kg; urine sodium 40 mmol/L), with normal thyroid function and random cortisol (Table 1). A non-contrast CT of the head showed no acute intracranial pathology but demonstrated chronic small-vessel ischaemic changes. In the context of hyponatraemia, these findings were consistent with hypotonic hyponatraemia. 

**Table 1:** Initial laboratory investigations on first admission (January 2024) TSH, thyroid-stimulating hormone; T4, thyroxine; CRP, C-reactive protein; ESR, erythrocyte sedimentation rate.

Investigation	Result	Reference range
Serum sodium	116 mmol/L	133–146 mmol/L
Serum osmolality	239 mOsm/kg	275–295 mOsm/kg
Urine osmolality	176 mOsm/kg	-
Urine sodium	40 mmol/L	-
Potassium	4.4 mmol/L	3.5–5.3 mmol/L
Urea	4.9 mmol/L	2.5–7.8 mmol/L
Creatinine	67 µmol/L	44–97 µmol/L
Adjusted calcium	2.37 mmol/L	2.20–2.60 mmol/L
Phosphate	0.80 mmol/L	0.80–1.50 mmol/L
Magnesium	0.60 mmol/L	0.70–1.00 mmol/L
Random cortisol	665 nmol/L	>450 nmol/L
TSH	1.53 mIU/L	0.35–5.50 mIU/L
Free T4	20.0 pmol/L	10.5–21.0 pmol/L
Serum glucose	6.5 mmol/L	3.9–7.8 mmol/L
Haemoglobin	110 g/L	118–158 g/L
Platelet count	265 × 10⁹/L	160–370 × 10⁹/L
Vitamin B12	539 ng/L	211–911 ng/L
CRP	<4 mg/L	0–9 mg/L
ESR	9 mm/hour	1–20 mm/hour
Lactate	2.11 → 1.11 mmol/L	0.36–1.39 mmol/L
Albumin	41 g/L	35–50 g/L
Total bilirubin	6 µmol/L	0–20 µmol/L
Alkaline phosphatase	72 U/L	30–130 U/L
Alanine transaminase	14 U/L	10–49 U/L

A detailed collateral history and medication review revealed an unintentional overdose of prescribed medications, primarily fluoxetine, with possible ingestion of her son's spironolactone. The patient had previously been prescribed fluoxetine 20 mg three times daily, which was later changed to a single 60 mg tablet once daily to reduce tablet burden. However, she continued taking the medication three times daily, resulting in a total daily dose of 180 mg. This supported the diagnosis of drug-induced SIADH secondary to fluoxetine overdose, which was considered the primary precipitant of her acute hypotonic hyponatraemia, with possible exacerbation by inadvertent spironolactone ingestion.

The endocrinology team reviewed the patient and concluded that this represented acute-on-chronic hyponatraemia, most likely secondary to drug-induced SIADH, with possible concomitant dehydration. They recommended a trial of an intravenous fluid challenge with close monitoring. The patient was commenced on cautious intravenous 0.9% sodium chloride. Her serum sodium initially improved to 119 mmol/L after 10 hours; however, it subsequently declined to 117 mmol/L the following morning, further supporting the diagnosis of SIADH. Following additional endocrinology input, fluid restriction to 1 L per 24 hours was instituted, with close monitoring and a plan for escalation to intensive care for hypertonic saline if clinically indicated. With fluid restriction, her serum sodium improved to 127 mmol/L the next day and reached 129 mmol/L prior to discharge. Fluoxetine was discontinued and replaced with mirtazapine following psychiatric review. She was discharged home five days after admission, clinically well, with community follow-up arranged.

Three days after discharge, she re-presented with behavioural changes, including leaving taps running, opening windows, preparing multiple meals without eating, and recurrent nausea and vomiting. Collateral history also described excessive thirst and increased water intake. Vital signs remained stable, and there was no evidence of acute infection. Repeat investigations demonstrated persistent hyponatraemia, with a serum sodium of 125 mmol/L. Iron studies performed for persistent anaemia confirmed iron deficiency.

Given her cachexia, weight loss, ongoing SIADH, and a history of previously documented right upper lobe ground-glass changes on imaging, a CT scan of the chest, abdomen, and pelvis was performed to investigate potential underlying causes of SIADH. This demonstrated a right upper lobe pulmonary nodule suspicious for stage IA1 lung adenocarcinoma (Figure 1). A subsequent PET-CT was consistent with radiological stage IA1 (T1miN0/1M0) lung adenocarcinoma. The probability of malignancy was estimated using the Herder model, a validated clinical prediction model that combines clinical and radiological characteristics with fluorodeoxyglucose (FDG) PET avidity to estimate the likelihood of malignancy in pulmonary nodules. The model estimated a 75% probability of malignancy for the pulmonary nodule, representing a high probability of lung cancer. Endobronchial ultrasound (EBUS)-guided lymph node biopsy demonstrated no malignant cells. Consequently, the diagnosis and subsequent management were based on the radiological findings rather than histological confirmation, and she underwent stereotactic ablative radiotherapy with outpatient follow-up.

**Figure 1:**
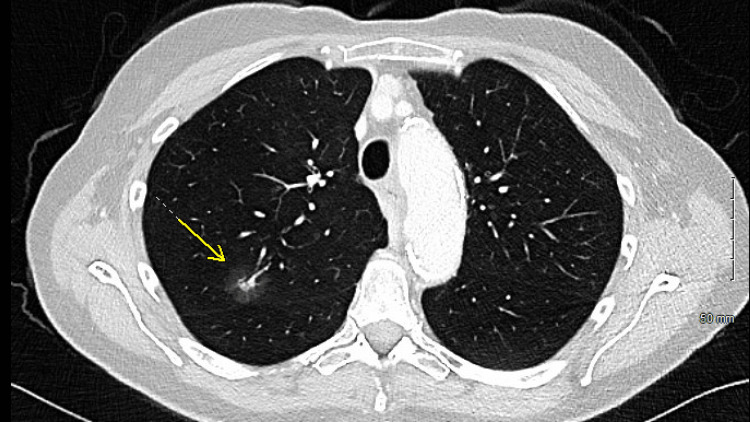
Selected axial contrast-enhanced CT image of the chest demonstrating a part-solid right upper lobe pulmonary nodule (yellow arrow) with a 7 mm solid component and a 20 mm ground-glass component, subsequently confirmed as stage IA1 lung adenocarcinoma. Yellow arrow: Right upper lobe pulmonary nodule.

Outpatient endoscopic investigation of her iron deficiency anaemia did not identify a significant gastrointestinal source. She was discharged with a diagnosis of persistent SIADH and advised to continue fluid restriction.

A few months later, the patient had three further admissions with recurrent confusion, hallucinations, behavioural disturbance, excessive thirst, and intermittent nausea and vomiting. Repeat biochemical investigations demonstrated persistent hypotonic hyponatraemia (Table 2), with only transient resolution in early November before subsequent deterioration. A repeat CT head demonstrated no acute changes.

**Table 2:** Serial biochemical investigations demonstrating recurrent hypotonic hyponatraemia Reference ranges: Serum sodium: 133–146 mmol/L; Serum osmolality: 275–295 mOsm/kg.

Date	Serum sodium (mmol/L)	Serum osmolality (mOsm/kg)	Urine osmolality (mOsm/kg)	Urine sodium (mmol/L)
November 2024	135	—	—	—
November 2024	124	270	162	35.1
December 2024	129	268	275	18.8

During a more detailed psychiatric assessment, it emerged that the patient had been a chronic cannabis user for over 25 years and had significantly increased her consumption following her bereavement and ongoing psychosocial stressors. She also reported switching from dried cannabis to cannabis resin, a more concentrated and potent formulation. This was felt to be a significant contributor to her recurrent neuropsychiatric presentations. Furthermore, her excessive thirst and fluid intake raised the possibility of cannabis-associated psychogenic polydipsia, or cannabis-related thirst with excessive free-water intake, contributing to persistent hyponatraemia. Additionally, her recurrent episodes of nausea and vomiting raised the possibility of cannabinoid hyperemesis syndrome, which may have further exacerbated her sodium imbalance. Liaison psychiatry assessment identified prolonged grief disorder and ongoing depression. Grief counselling and referral to drug and alcohol services were offered. However, the patient declined referral on multiple occasions, stating that cannabis use remained her primary coping mechanism for ongoing stress and multiple bereavements. She was ultimately discharged with a dosette box to support medication adherence and reduce the risk of recurrent medication errors. She was advised to continue fluid restriction, with ongoing outpatient follow-up arranged.

## Discussion

SIADH is one of the most common causes of hyponatraemia, accounting for approximately one-third of all cases [2]. Drug-induced SIADH typically results in a gradual decline in serum sodium and is often clinically well tolerated until hyponatraemia becomes severe [4]. However, hyponatraemia is frequently multifactorial, and in patients receiving long-term psychotropic medications, clinically significant sodium derangements may only emerge when additional precipitating factors are present [4,5]. In our patient, several recognised risk factors for SIADH were present, including advanced age (>60 years), female sex, baseline hyponatraemia (<135 mmol/L), selective serotonin reuptake inhibitor (SSRI) use, and smoking [4].

The patient initially presented with profound hypotonic hyponatraemia in the setting of an unintentional SSRI overdose, in this case fluoxetine. Initial biochemical investigations were consistent with SIADH, a recognised complication of SSRI therapy [4,6]. Given the temporal relationship between the overdose and the onset of hyponatraemia, the initial presentation was attributed to SSRI-induced SIADH. Drug-induced SIADH usually resolves within days to weeks after discontinuation of the medication [6]. However, despite discontinuation of fluoxetine and replacement with mirtazapine, she continued to experience recurrent episodes of confusion and hyponatraemia.

Approximately 10 months after her initial presentation, she re-presented with recurrent confusion and persistent hypotonic hyponatraemia, with a serum sodium of 124 mmol/L, serum osmolality of 270 mOsm/kg, and urine osmolality of 162 mOsm/kg. This persistence raised suspicion for an alternative or additional underlying pathology. Subsequent investigations led to the diagnosis of lung adenocarcinoma. Although SIADH has been reported in association with non-small-cell lung cancer, this remains an uncommon phenomenon and is described predominantly in isolated case reports [7]. Given the rarity of this association and the patient's initial SSRI overdose, her malignancy was not considered the primary driver of her recurrent presentation with acute-on-chronic hyponatraemia.

The patient, however, was noted to smoke large amounts of cannabis and had switched from herbal cannabis to cannabis resin (a cannabis concentrate). Cannabis concentrates contain substantially higher concentrations of delta-9-tetrahydrocannabinol (THC), the principal psychoactive constituent of cannabis, than herbal cannabis. The National Institute on Drug Abuse (NIDA) reports that cannabis concentrates commonly contain 40%-90% THC, compared with considerably lower concentrations in herbal cannabis [8]. This raised the possibility of psychogenic polydipsia versus cannabis-related thirst with excessive free-water intake. In addition, her recurrent nausea and vomiting raised the possibility of cannabinoid hyperemesis syndrome.

Psychogenic polydipsia, a form of primary polydipsia, is classically defined as a compulsive drive to consume excessive amounts of water and occurs most commonly in patients with psychiatric disorders, particularly schizophrenia [9]. It is typically associated with maximally dilute urine, often with a urine osmolality below 100 mOsm/kg [4]. This patient's biochemical profile was not consistent with isolated primary polydipsia. Her persistently elevated urine osmolality suggested ongoing antidiuretic hormone activity, supporting an underlying diagnosis of SIADH. There is also limited evidence in the literature directly linking cannabis use to psychogenic polydipsia [10]. This, therefore, could suggest cannabis-related thirst with excessive free-water intake as the more likely contributing factor. Cannabis is known to cause xerostomia and thirst, and case reports have described severe hyponatraemia following excessive water consumption after cannabis use [10]. In this patient, excessive free-water intake may have exacerbated the degree of hyponatraemia in the setting of underlying SIADH rather than representing isolated primary polydipsia.

## Conclusions

This case highlights the importance of reconsidering the differential diagnosis in patients with persistent or recurrent hyponatraemia despite removal of an apparent precipitating factor, as well as ensuring adherence to fluid restriction. Although the initial presentation was consistent with SSRI-induced SIADH, the persistence of hypotonic hyponatraemia well beyond the expected period of resolution prompted further investigation, ultimately revealing possible additional contributing factors.
